# Post-Traumatic Stress Disorder 4 Years after the COVID-19 Pandemic in Adolescents with Different Levels of Physical Activity Engagement: A Repeated Cross-Sectional Study

**DOI:** 10.3390/ijerph21080975

**Published:** 2024-07-26

**Authors:** Giulia Di Martino, Marco Centorbi, Andrea Buonsenso, Giovanni Fiorilli, Carlo della Valle, Giuseppe Calcagno, Enzo Iuliano, Alessandra di Cagno

**Affiliations:** 1 Department of Medicine and Health Sciences, University of Molise, 86100 Campobasso, Italy; giulia.dimartino21@gmail.com (G.D.M.); marco.centorbi@hotmail.it (M.C.); andreabuonsenso@gmail.com (A.B.); fiorilli@unimol.it (G.F.); giuseppe.calcagno@unimol.it (G.C.); 2 Department of Neurosciences, Biomedicine and Movement, University of Verona, 37314 Verona, Italy; 3 Faculty of Medicine, University of Ostrava, 73000 Ostrava, Czech Republic; enzo.iuliano84@gmail.com; 4 Faculty of Psychology, eCampus University, 22060 Novedrate, Italy; 5 Department of Movement, Human and Health Sciences, University of Rome “Foro Italico”, 00135 Rome, Italy; alessandra.dicagno@uniroma4.it; 6 Department of Human Sciences, Guglielmo Marconi University, 00193 Rome, Italy

**Keywords:** avoidance, intrusion, psychological distress, psychological issues, COVID-19 restrictions, sport, IES-R questionnaire, adaptation process

## Abstract

The aim of this study was to assess whether the psychological impact of the COVID-19 pandemic on children and adolescents had decreased four years after the initial assessment. This study aimed to determine if children with an active lifestyle and participation in sports activities were protected against this traumatic stress. This study included a total of 284 Italian participants assessed at two different time points: the first assessment was conducted in 2020 when the children were aged 9–12 years, and a second assessment was carried out four years later when the participants were aged 13–16. Participants completed the Impact of Event Scale–Revised questionnaire (IES-R), with the IES-8 and IES-15 versions used accordingly based on age group. In the 2020 assessment, 146 (51.4%) reported a score higher than the cut-off for significant traumatic stress, while in 2024, only 49 participants (17.2%). The chi-square analysis indicated that this decrement was statistically significant (*p* < 0.001). RM-ANOVA showed a significant reduction for both Intrusion Score and Avoidance Score (*p* < 0.001). A statistical interaction between gender and time was observed. There were weak correlations between the level of children’s sport practice, and no differences between those who engage in individual or team sports. Despite this study showing that young people are overcoming the pandemic crisis and its consequences, identifying potential modifiable risk factors and empowering protective factors remains crucial, especially for those who continue to experience psychological issues. The restrictions particularly impacted active children by disrupting their routine, which may have compromised the universally recognized protective value of sports.

## 1. Introduction

The 2019 coronavirus (COVID-19) outbreak caused home confinement. After the virus began to spread, the Italian Prime Minister mandated the enforcement of physical distancing measures. This led to the closure of schools and numerous recreational, cultural, and sports centers, aimed at preventing transmission between individuals. The resulting prolonged inactivity and lack of in-person interactions could have negatively affected children and adolescents. There is a growing global awareness regarding the persistent psychological consequences induced by the COVID-19 pandemic, supported by empirical evidence [1]. Children and young people are potentially vulnerable to the emotional impact of traumatic events that have disrupted their daily routines [2]. Especially during adolescence, a critical phase of psychosocial development where signs of mental health issues might emerge, young individuals may be especially vulnerable to experiencing negative psychological effects due to the COVID-19 pandemic and its consequences [3,4]. However, the long-term implications of psychological symptoms related to infection and/or periods of social distancing are still unclear [5].

The lockdown caused by COVID-19 has been particularly high-risk in generating long-term psychological distress in adolescents and children [6]. Recent scientific research showed that anxiety or depression symptoms were found in as many as 49.5% for anxiety and 63.8% for depression, among the evaluated children and adolescents [7]. Additionally, irritability and anger are frequently reported, with a percentage of 51.3% among the youth in the examined sample [7].

Engagement in physical activity and sports before, during, and after the spread of COVID-19 influences the critical outcomes post COVID-19, ensuring benefits for physical and mental health [8]. Social connections promoted by physical activity improve children’s and adolescent wellbeing [9,10]. Extra-curricular sports activities counteract the suspension of social connection due to long-term COVID-19 measures for health [11].

This study aimed to assess whether the psychological impact of the COVID-19 pandemic on children and adolescents had decreased four years after the initial outbreak, while also investigating the coping strategies adopted by participants who were children during the pandemic and later became adolescents. Coping strategies refer to a sequence of actions or cognitive processes employed when encountering a challenging or unpleasant situation, aimed at managing or altering one’s response to it [12]. These strategies generally involve a deliberate and direct approach to addressing problems, such as defense mechanisms [13]. Moreover, this study aimed to determine if children with an active lifestyle and participation in sports activities were somehow protected against this traumatic stress and could more easily overcome the psychologically traumatic impact of the pandemic.

The current study aimed to answer the following three questions: (1) How has the COVID-19 lockdown influenced the long-term wellbeing of children and adolescents?; (2) Which coping strategies did children and adolescents adopt during the COVID-19 pandemic and four years after it?; (3) Could an active lifestyle and participation in sports activities have represented a protective factor against long-term psychological issues and distress in children and adolescents?

## 2. Materials and Methods

### 2.1. Study Design

This research is a longitudinal study. Specifically, a survey was administered to groups of children and adolescents in 2020 (during the pandemic) and again in 2024 (currently). The questionnaire investigated stressful life events and served as a measure of post-traumatic stress disorder symptoms.

### 2.2. Participants

The participants in this study (284 children aged between 9 and 12 years in 2020 and aged between 13 and 16 years in 2024) were recruited using convenience samples from primary and secondary schools in Italy during 2020. Regarding the geographic area of the first sample in 2020, it was performed in the northern, central, and southern areas of Italy. In 2024, the same subjects were recruited again, while participation in the second recruitment primarily involved central and northern Italy. To be enrolled in this study, participants had to meet the following inclusion criteria: (a) aged between 9 and 12 years in 2020. The exclusion criteria were (a) a not healthy status and (b) using medications that could affect their psychophysiological condition.

Informed consent was obtained from the parents of the participants via email, who then administered the questionnaire on behalf of their children.

This study was designed and conducted following the Helsinki Declaration. This study was approved by the local ethical committee of the University of Rome “Foro Italico” (178/2023). The characteristics of the sample are reported in Table 1.

### 2.3. Procedures

To assess COVID-19 and the consequent quarantine psychological impact in Italy, a national survey on children and adolescents was carried out in two different periods: a first period (from the beginning of April to May 2020) and a second period (from the beginning of February to the end of April 2024).

An informative letter reporting the main purpose of this study related to the perceived stress following the COVID-19 pandemic was sent via email to several Italian schools in 2020, and successively, the questionnaires were sent directly to the students in 2024.

Before the questionnaire, a cover letter explaining the nature of the research, including assurance of confidentiality and anonymity, was added. Personal data were collected anonymously through the creation of a personal security code.

Both the 2020 and 2024 questionnaires were structured as follows: the first section of the survey aims to collect sociodemographic information, including gender, age, region of residence, type of sport practised, if any, and years of training experience, assessed by weekly training volume (in minutes per week). The second section focused on assessing psychological distress using different versions of the Impact of Event Scale–Revised questionnaire tailored to participants’ age groups: the IES-8 was used in 2020, while the IES-15 was used in 2024.

### 2.4. Screening Questionnaire

To measure current subjective distress, the Impact of Event Scale–Revised (IES-R) was used. The questionnaire was designed to measure post-traumatic stress disorder (PTSD) symptoms, and it is a short, easily administered self-report questionnaire. Traumatic events include not only those that directly threaten life or physical integrity but also those perceived as such by individuals due to specific characteristics such as negative valence, unpredictability, and lack of control.

Two different versions, validated in Italy [14,15], were used according to the population age: children underwent the Impact of Event Scale-8 (IES-8) questionnaire [16], and Teenagers underwent IES-15 [17] to ensure adequate understanding across different age groups. Each version is structured with Likert-scale scoring: 0 for never, 1 for sometimes, 3 for rarely, and 5 for often. A cut-off score was set to 30 for the IES-15 and 16 for the IES-8 according to the survey instructions [16,17]. Moreover, the questionnaire analyses two different subscales: Intrusion and Avoidance.

As the two questionnaires administered to younger children (IER-S 8 item) and older adolescents (IER-S 15 item) generated scores on different scales (respectively, 0–40 for IER-S 8 and 0–75 for IER-S 15, where higher scores indicated a worse situation), the scores were normalised to percentage scores using the formula [score obtained/maximum score × 100]. This normalisation ensured that each participant had a score ranging from 0% (best score) to 100% (worst score), independently from age and the questionnaire administered. The intrusion and avoidance scales were analysed as average scores per participant, with no differences for the two versions of the survey, as both had scores ranging from 0 (best score) to 5 (worst score). The subscales scores, Intrusion and Avoidance, indicate the possibility that these different behaviours occur. It has a good test–retest reliability (0.79 to 0.89) and satisfactory internal consistency (Cronbach’s α = 0.78 to 0.820) [18].

### 2.5. Statistical Analysis

The data are presented as mean and standard deviation. The Shapiro–Wilks test was used to assess the normal distribution of the data.

The number of participants scoring above the cut-off was counted for both the questionnaires administered in 2020 and those administered in 2024. These values were compared using Chi-squared analysis to assess whether the number of symptomatic/not symptomatic participants after 4 years significantly changed.

The scores from the survey administered in 2020 were compared with those from 2024 using paired analyses (RM-ANOVA test). The score obtained by the IES-R (TotScoreNorm), the intrusion subscale score (IntScore), and the avoidance subscale score (AvScore) were considered independent variables of the analysis.

The RM-ANOVA analysis was conducted 3 times: the first analysis involved all 284 participants. A subsequent analysis was performed on participants who scored above the cut-off in the 2020 assessment to understand if their scores changed over time, considering that in 2020, these individuals reported values of IES-R, suggesting PTSD. The last analysis was performed on participants who scored above the cut-off in 2020 and 2024.

Similarly, the volume of sports activities between 2020 and 2024 was compared. This comparison aimed to evaluate whether this parameter significantly changed over time. The analysis was conducted for all participants, for participants who scored above the cut-off in 2020, and for participants who scored above the cut-off in both 2020 and 2024.

Gender, age, and type of sport (individual or team sports) were considered in the analysis to evaluate whether these variables might have had a significant impact on the IES-R scores.

Finally, Pearson’s correlation analyses were conducted to examine the relationship between (1) training volume (expressed in minutes per week) and (2) years of experience in training vs. (1) TotScoreNorm, (2) IntScore, and (3) AvScore obtained by the IES-R survey to evaluate whether higher training volumes or longer training experience are positively or negatively correlated with PTSD.

A *p*-value < 0.05 was considered statistically significant. Data were analysed using the SPSS statistical software package (IBM, v.29.0, Chicago, IL, USA).

## 3. Results

In the 2020 assessment, 146 out of 284 participants (51.4% of the sample) reported a score higher than the cut-off, while in 2024, the number decreased to 49 out of 284 participants (17.2%). The chi-square analysis indicated that this decrement was statistically significant (χ^2^ = 73.48; *p* < 0.001).

The Shapiro–Wilks test indicated a normal distribution of the TotScoreNorm, IntScore, and AvScore variables. The results obtained by RM-ANOVA show that the participants significantly reduced their TotScoreNorm of IES-R after 4 years (F1,283 = 213.847; *p* < 0.001). A significant reduction over time was also observed in the IntScore (F1,283 = 382.121; *p* < 0.001) and in the AvScore (F1,283 = 30.964; *p* < 0.001).

As previously stated, in 2020, 146 of the 284 participants obtained a score above the cut-off in the IES-R survey. The RM-ANOVA conducted on these 146 participants revealed a significant reduction in the TotScoreNorm after 4 years (F1,145 = 440.546; *p* < 0.001); also, in this case, a significant reduction over time was obtained in the IntScore (F1,145 = 641.965; *p* < 0.001) and in the AvScore (F1,145 = 68.067; *p* < 0.001).

Among these 146 participants, 49 participants maintained a score above the cut-off, even in the 2024 assessment. This implies that 17,2% of the total sample (49 of 284 participants) continue to show symptoms of PTSD after 4 years. The RM-ANOVA conducted on these 49 participants revealed a significant reduction over time in the TotScoreNorm (F1,48 = 24.272; *p* < 0.001) and in the IntScore (F1,48 = 57.557; *p* < 0.001); however, no significant change was observed in the AvScore (F1,48 = 0.157; *p* = 0.694).

The time spent on physical activity or sports between 2020 and 2024 (measured in minutes per week) showed no statistically significant differences between the two time points. This significant difference was observed for all 284 participants (F1,283 = 0.425; *p* = 0.515), for the 146 participants who scored above the cut-off in 2020 (F1,145 = 0.023; *p* = 0.880), and for the 49 participants who scored above the cut-off in both 2020 and 2024 (F1,48 = 3.623; *p* = 0.063).

All the results are reported in Table 2.

The analyses showed that there were no statistically significant differences between females and males (*p* = 0.537). However, a statistical interaction Gender*Time was observed, with a significantly favourable trend for males, who showed a higher reduction over time compared with females (F1,283 = 8.504; *p* = 0.004).

No statistically significant differences were found for age or type of sport.

Pearsons’ analysis showed negative but very weak correlations between both training volume and years of experience vs. TotScoreNorm, IntScore, and AvScore both in the data collected in 2020 and 2024 (r ranged from −0.025 to −0.183).

## 4. Discussion

The total score of the IER-S test administered to children during the pandemic is significantly higher compared with the score achieved by the same subjects four years after the pandemic. It appears that the children are overcoming the stress from the traumatic event, both from the pandemic itself and its consequences.

However, the results indicating that the cut-off for the total score still reveals that 49 children have not overcome the psychological issues caused by COVID-19, highlighting the need for targeted interventions. Few studies have reported that psychological distress symptoms have persisted without significant improvement from the onset to the later stages of the pandemic [19]. The individual reactions to the COVID-19 breakout and the successive periods have been substantially different. Children and adolescents have been at high risk for suffering from psychological disorders as a consequence of the pandemic restrictions [3]. Several studies have identified poor sleep and nightmares, physical discomfort, agitation, inattention, clinginess, and separation issues as the most frequent distress symptoms in children [20,21].

A particularly interesting finding was the evidence that children who never overcame the level of distress alarm (49 subjects) and exhibited greater use of intrusion-oriented coping strategies during the pandemic are more likely, four years later, as adolescents, to utilise avoidance strategies, tending to distance themselves from stressful situations and engaging in substitute activities [22]. Drawing from earlier research [23], we hypothesised that employing positive coping methods (such as positive representation) would correlate with lower perceived stress levels, while the use of negative coping strategies (such as intrusion) would lead to higher perceived stress levels in children. The pandemic and its restrictions in the initial period were unexpected, acting as a sudden negative and uncontrollable event, so it is understandable that children’s reactions exceeded the threshold for normal distress levels. Symptoms of re-experiencing unpleasant and intrusive memories in the form of images, thoughts, or perceptions have characterised the first time of the coping reaction of children to pandemic restrictions and seem to be the first way to react [24]. The acute stressful event led to increased activity of the noradrenergic system, which can contribute to re-experiencing symptoms. At follow-up, after four years, the event is no longer unexpected, and a higher number of avoidance symptoms were frequently observed [25]. Avoidance serves as a defensive mechanism against issues, where individuals avoid encountering feared situations or things to live in a normal way [26].

No significant differences in overcoming distress levels in the sample were found when diving by gender; however, significant gender/time interactions were found. Males tend to have a better trend of overcoming psychological distress, probably due to their less emotive psychological status.

There were weak correlations between the level of children’s sport practice, and no differences between those who engage in individual or team sports. A plausible explanation is that not active children likely did not substantially change their lifestyle during the home confinement due to social restrictions imposed by the COVID-19 pandemic [27]. However, for active children, the COVID-19 outbreak altered many regular aspects of life, including exercise routines and sports activities [28]; also, after the resumption of activities, they had to adapt to new rules and habits. Children who consistently participated in sports, making notable changes to their weekly schedules, sacrificed activities that contributed to their physical and emotional health [29]. The protective role of physical and sports activities, which we expected to serve as a coping mechanism against the psychological disorders associated with the pandemic, may have been counterbalanced by the forced cessation of their preferred activities due to the circumstances. The perception of isolation and the loss of sportive milestones (results of sports competitions and events and sports exams) have impacted more than their no active counterparts, for whom lifestyles essentially remained unchanged [6]. Even the sharing of activities, expectations, and adversity typical of people engaged in a team sport did not prove to be a protective factor, as emerged from previous studies [30]. Probably, the young age of the children did not allow for frequent contact among teammates, which at this age is mediated by parents. Furthermore, the reduced level of weekly physical activity and the increased time spent engaged in screen usage may have increased the stress perception (IER-S) between the individual and team sports performers [20].

Despite previous findings, no significant differences in gender-related perceived stress and emotional response behaviours were found either during or after the pandemic [31,32]. Women, on the other hand, generally show a more emotional reaction to events affecting their lives. However, a significant Gender*Time interaction emerged, indicating a trend where males showed better resilience in overcoming several issues, possibly due to the young age of children in the initial investigation and the less pronounced gender differences at that time.

## 5. Conclusions

In conclusion, to answer the first research question of this paper, we can affirm that despite young people overcoming the crisis caused by the pandemic, a small number of the sample did not overcome their psychological issues. For this reason, it is fundamental to identify potential but modifiable risk factors to address and protective factors to empower.

In response to the second question, we noted that coping strategies changed over time: during the COVID-19 pandemic and social restrictions, children reacted with intrusions, likely a coping strategy exacerbated by the lack of social interactions, while in the subsequent period, probably, the intention to remove unpleasant sensations highlighted an increase in avoidance.

For the third question, we can answer that despite no results emerging in this survey enhancing communication, engaging in collaborative games, increasing physical activity, reducing time spent playing video games, or using technological tools to communicate can be effective solutions to alleviate the emotional and psychological distress caused by the prolonged quarantine period and its long-term consequences. 

## 6. Strength and Limitation

The main strength of this study lies in the follow-up of the results 4 years after the initial investigation to assess the trajectory of the patients’ psychological experience related to COVID-19 and its consequences. The longitudinal design provides the opportunity for interesting analyses; however, it does not allow for a large sample size. The symptomatology and level of PA were subjectively and retrospectively assessed, which could raise concerns about memory bias; moreover, the responders are children and adolescents, and it is possible that their text comprehension was not always correct. Another limitation regards the lack of information about the health status related to the COVID-19 infection of participants, concerning specific comorbidity and/or pre-existing chronic disease. Consequently, as an exclusion criterion, the sample did not include participants with clinical issues. Furthermore, our sample relied on participants’ self-report and could be affected by recall bias, especially due to their young age. Further studies could address the limitations of the present study.

## Figures and Tables

**Table 1:** Characteristics of the sample.

Characteristic of the Sample	Variables
Number of participants	284 (150 male, 154 females)
Age in 2020 (years)	10.85 ± 1.05 (min 9; max 12)
Volume of training in 2020 (minutes/week)	394.65 ± 309.97 (min 60; max 1440)
Volume of training in 2024 (minutes/week)	429.93 ± 267.50 (min 120; max 1440)
Experience in training in 2020 (years)	4.33 ± 2.28 (min 0; max 7)
Experience in training in 2024 (years)	6.02 ± 2.99 (min 0; max 10)

**Table 2:** Result of the RM-ANOVA.

Sample	IES-R in 2020	IES-R in 2024	*p*-Value
All participants (*n* = 284)			
TotScoreNorm (%)	42.83 ± 19.19%	22.86 ± 15.74%	*p <* 0.001 *
IntScore (Likert Score 0–5)	2.42 ± 1.23	0.91 ± 0.80	*p <* 0.001 *
AvScore (Likert Score 0–5)	1.88 ± 1.17	1.38 ± 0.99	*p <* 0.001 *
Weekly training volume (min/week)	407.46 ± 308.44	423.03 ± 258.46	*p =* 0.515
Participants with score over the cut-off in 2020 (*n* = 146)			
TotScoreNorm (%)	58.20 ± 12.39%	26.72 ± 18.49%	*p <* 0.001 *
IntScore (Likert Score 0–5)	3.25 ± 0.94	1.09 ± 0.94	*p <* 0.001 *
AvScore (Likert Score 0–5)	2.57 ± 1.05	1.58 ± 1.09	*p <* 0.001 *
Weekly training volume (min/week)	411.64 ± 313.61	417.05 ± 296.04	*p =* 0.880
Participants with score over the cut-off both in 2020 and 2024 (*n* = 49)			
TotScoreNorm (%)	58.62 ± 13.65%	48.03 ± 0.08%	*p <* 0.001 *
IntScore (Likert Score 0–5)	3.27 ± 0.98	2.13 ± 0.66	*p <* 0.001 *
AvScore (Likert Score 0–5)	2.59 ± 1.02	2.66 ± 0.70	*p =* 0.694
Weekly training volume (min/week)	346.12 ± 171.43	424.29 ± 235.16	*p =* 0.063

* Statistically significant.

## Data Availability

Data are available under request to the corresponding author.
